# Nutritional ingredients and prevention of chronic diseases by fermented koumiss: a comprehensive review

**DOI:** 10.3389/fnut.2023.1270920

**Published:** 2023-10-19

**Authors:** Weikang Xue, Xiaoxia Yuan, Zhaojun Ji, Hua Li, Yitong Yao

**Affiliations:** College of Life Science and Food Engineering, Inner Mongolia Minzu University, Tongliao, Inner Mongolia, China

**Keywords:** koumiss, lactic acid bacteria, nutritional ingredients, chronic disease prevention, *Saccharomyces*

## Abstract

Koumiss, a traditional fermented dairy product made from fresh mare milk, is a sour beverage that contains an abundance of microbial communities, including lactic acid bacteria, yeast and others. Firstly, probiotics such as *Lacticaseibacillus* in koumiss can induce the secretion of immunoglobulin G in serum and interleukin-2 in the spleen while beneficial *Saccharomyces* can secrete antibacterial compounds such as citric acid and ascorbic acid for specific immunopotentiation. Additionally, more isoflavone in koumiss can regulate estrogen levels by binding to its receptors to prevent breast cancer directly. Bile salts can be converted into bile acids such as taurine or glycine by lactic acid bacteria to lower cholesterol levels *in vivo*. Butyric acid secretion would be increased to improve chronic gastrotis by regulating intestinal flora with lactic acid bacteria. Finally, SCFA and lCFA produced by *Lacticaseibacillus* inhibit the reproduction of pathogenic microorganisms for diarrhea prevention. Therefore, exploring the mechanisms underlying multiple physiological functions through utilizing microbial resources in koumiss represents promising avenues for ameliorating chronic diseases.

## Introduction

Koumiss, with a long history, is one of the favored traditional dairy products among nomadic people living in the grasslands of northern China. It is a fermented beverage and is also known as *kumiss*, *chigo*, *chige*, *arrag*, or *airag*. It has an acidic-alcoholic profile, a milky white appearance, and a subtle aroma and flavor. Koumiss is usually fermented naturally using traditional methods with fresh mare milk and can provide abundant nutrients for drinkers from June to September in Inner Mongolia, Xinjiang, Tibet, and other provinces in China (1–3). Traditional methods of making koumiss using natural fermentation are described here. Fresh mare milk is filtered and cooled to approximately 20°C; it is subsequently poured into a wooden barrel, leather bag, or plastic barrel that contains some koumiss residue as a fermentation starter. Next, a wooden stick is used to stir the fresh mare milk violently to form a mixture that then ferments naturally at ambient temperature for 1–3 days (4). The yeast, lactic acid bacteria, other microorganisms, and alcohol content are determined by fermentation time. Notably, the stirring operation is very important for homogenization during fermentation to allow for the more uniform distribution of acidity and to break casein clots in the gel formation of proteins. Finally, koumiss with good consistency and structure, as well as delicate taste, are obtained (5, 6). Therefore, the short shelf life of koumiss, only 3–5 days, coupled with the relatively low milk output of mares, has restricted opportunities for large-scale industrial production and consumer acceptance. However, the complex microbial communities present in koumiss, including benificial lactic acid bacteria, yeast, etc., have been found to help regulate gut microbiota, metabolites, and host immunity (5, 7). Koumiss also possesses preventive and therapeutic properties against chronic diseases such as pulmonary tuberculosis, hyperlipidemia, and cardiovascular and cerebrovascular diseases (5, 8, 9). At the same time, some potential harmful bacteria still survives in the fermentated koumiss.

## Nutritional ingredients of koumiss

### Fresh mare milk

Fresh mare milk with particular composition, hypoallergenicity, and nutraceutical properties (10), the raw material of koumiss, contains many nutrients essential for the human body. These include proteins, fats, lactose, galactose, vitamins, enzymes, and minerals, etc. (Table 1). The nutrients in mare milk are comparable to those found in human breast milk (HBM) and can be used as a substitute for infant formula (1, 11). Additionally, the contents of essential amino acids, unsaturated fatty acids, and lactose which can promote calcium absorption among humans, are greater in mare milk than in other domestic animal milks (12). Furthermore, fresh mare milk is more easily digested and absorbed by the human body. This is because digestible whey protein constitutes approximately 40% of the total protein, more than twice as much as found in cow milk. The indigestible casein is also relatively lower in contents (13). The lower fat content and smaller fat droplets in fresh mare milk are more easily absorbed by the human body (14, 15).

**Table 1:** The nutrient composition of koumiss.

Nutrients	Contents	Nutrients	Contents	Nutrients	Contents
Protein	1.80–2.26%	Lactose	2.58–4.30%	^V^_C_	1.76–5.79 mg/100g
Fat	0.60–2.20%	Lactic acid	0.70–9.00%	^V^_E_	19.00–99.80 μg/100g
Lipid	0.60–1.30%	Ethanol	0.60–13.68%	^V^B1	4.14–9.00 μg/100g
Ash content	0.39–0.50%	Carbon dioxide	0.50–0.90%	^V^B2	5.06–100.00 μg/100g
Total solids	10.60–11.30%	Titratable acidity	98.63±3.25(°T)	Folic acid	10.97 μg/100g

Fresh mare milk is rich in vitamins C, A, E, D, B_1_, B_2_, B_12_, and calcium, phosphorus, and other mineral elements, of which the ratio of calcium and phosphorus is 2:1 which is very similar to HBM (16). Mare milk also contains large numbers of essential trace elements such as Zn, Cu, V, Cr, Ni, Co, Mo, and other elements not essential but beneficial to the human body, such as Sr., Rb, Ba, and Li (11). An additional twelve rare elements that positively affect the human body have also been found in mare milk. The contents of rare elements from high to low are Sc, Ce, Nd, La, Y, Sm, Eu, Pr, Gd, Yb, Er, and Ho (11). Additionally, the content of lysozyme in mare milk is twice that of HBM and promotes recovery from gastric ulcers and upper respiratory infections, as well as healing wounds and postoperative scars. The combination of lysozyme and lactoferrin in mare milk is also a type of natural anti-infection substance with bactericidal effects (5, 17, 18).

### Koumiss

The abundant nutrients and bioactive compounds found in koumiss are also metabolized by probiotic lactic acid bacteria, yeast, and other microorganisms in addition to those found in fresh mare milk. Unique tastes and high nutritional values in koumiss are the results of enzyme catalysis by lactic acid bacteria and yeast, such as the metabolism of carbohydrates and amino acids and the biosynthesis of fatty acids, which all benefit the pancreas and promote digestion (11, 12, 19). Gastrointestinal motility disorders can also be relieved because the acid amines and peptones converted from casein and albumin in koumiss can be absorbed easily and quickly by patients (13). Whey proteins rather than caseins are major proteins in the koumiss (20). Additionally, a variety of volatile organic compounds in koumiss, including lactic acid, ethanol, carbon dioxide, and other substances converted from lactose by lactic acid bacteria and yeast would prove beneficial for consumers who are lactose intolerant (Table 1) (3, 12). Essential fatty acids, such as α-linoleic acid and linolenic acid, are higher in koumiss than in other unsaturated fatty acids and can also be produced through natural fermentation (21).

Furthermore, organic acids, aromatic compounds, extracellular polysaccharides, peptides, enzymes, bacteriocins, and other special nutrients are also produced during natural fermentation. The organic acids and bacteriocins inhibit harmful microorganisms, and bioactive peptides have excellent anticancer and antioxidant properties (5, 12). Therefore, the high nutritional values and rich functional components identified in koumiss are primarily the results of the original fresh mare milk and microbiological metabolites found in it (Table 1) (11, 22).

## Prevention of chronic diseases by drinking koumiss

### Koumiss can effectively improve specific immunopotentiation

**Figure 1:**
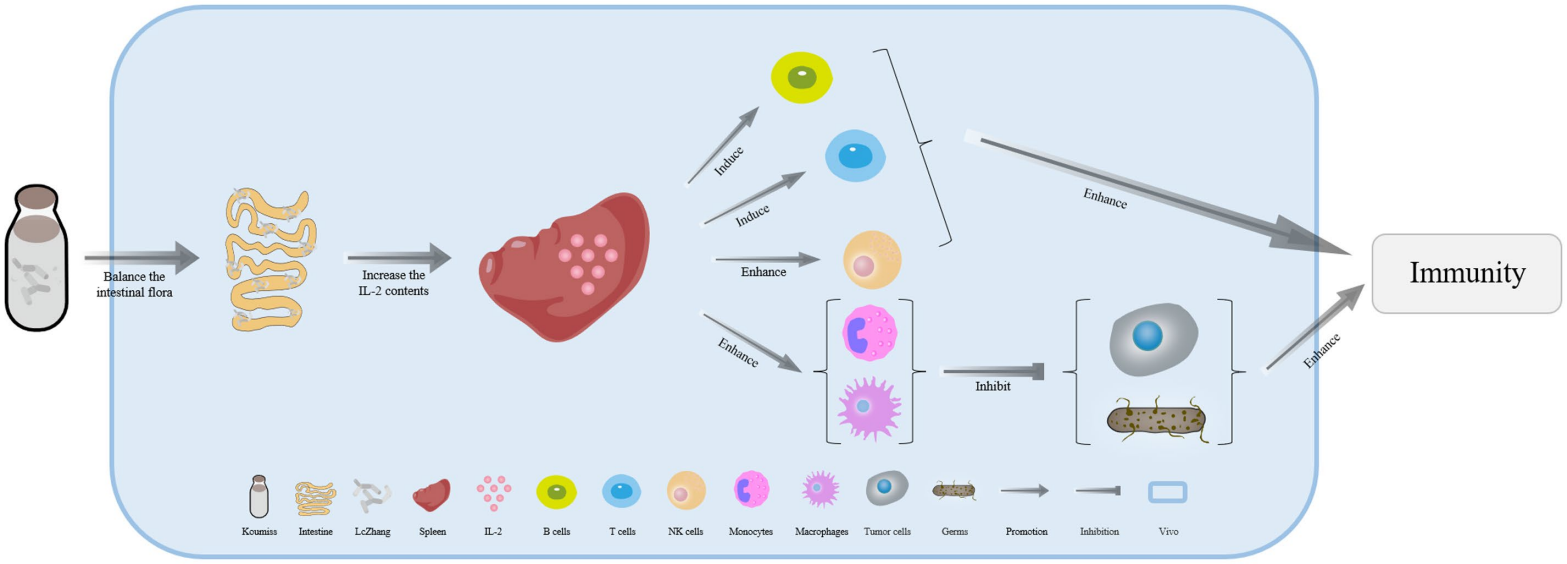
The mechanism of immunopotentiation in various ways with koumiss. Drinking koumiss with *L. casei* Zhang can induce the secretion of interleukin-2 (IL-2) in the spleen, which in turn increases the activation of T and B lymphocytes, enhances NK cell activity, and ultimately enhances immunopotentiation. Furthermore, the increased content of IL-2 in the spleen enhances the activation of monocytes and macrophages against tumor cells and bacteria.

Numerous probiotics found in koumiss, especially lactic acid bacteria, can directly regulate the intestinal flora balance and microenvironment. As a result, the probiotics and their metabolites can improve specific immunopotentiation (23–25). *Lacticaseibacillus casei* Zhang isolated from koumiss can effectively inhibit the inflammatory response caused by polyinosinic:polycytidylic acid (Poly I:C). Poly I:C is often used as an analog double-stranded RNA (dsRNA) virus to simulate the viral infection process in macrophage cells (RAW264.7) (26). Research has shown that *L. casei* Zhang in koumiss can prevent or mitigate inflammatory reactions by reducing the amount of tumor necrosis factor-α (TNF-α) in serum (27, 28). Additionally, *L. casei* Zhang contributes to immunopotentiation by increasing the level of total immunoglobulin G (IgG) in serum and the content of interleukin-2 (IL-2) in the spleen. This induces the activation of T and B lymphocytes, enhances NK cell activity, and increases the activity of monocytes and macrophages against tumor cells or bacteria (Figure 1) (29–31). *L. casei* Zhang can stimulate the activation of T helper cells and NK cells, which then secrete the proinflammatory cytokine interferon γ (IFN-γ). IFN-γ can bind to the transmembrane glycoprotein receptor IFN-γR, inducing macrophage cells and T lymphocytes to flow to the inflammatory site and enhance immune reactions (28, 32, 33). Another strain isolated from koumiss, *Lactobacillus acidophilus* NCFM, can increase the murine dendritic cell expression of interferon β (IFN-β), interleukin-12 (IL-12), and interleukin-10 (IL-10). These observations demonstrate the strong antiviral ability of koumiss (26, 34).

Drinking koumiss which contains *L. casei* Zhang can promote the transcription of toll-like receptor 3 (TLR3) for double-stranded ribonucleic acid (dsRNA) in the pattern recognition receptor family. This is located on the surface of epithelial cells. Consequently, TLR3 upregulates the expression of proinflammatory factors by recognizing ligands and activates the nuclear factor kappa B (NF-κB) signaling pathway, thereby effectively promoting immune responses (26, 35). Additionally, the transcription of the immune receptor TLR9 by *L. casei* Zhang present in koumiss can activate plasmacytoid dendritic cells (pDCs) and B lymphocytes, thus enhancing immunological effects when the human body is stimulated by harmful bacteria carrying common unmethylated CpG motifs in their genomes (26, 36, 37).

Consuming koumiss probiotics has been shown to increase the expression of peroxisome proliferator-activated receptor α/β/δ (PPAR-α/β/δ) in mice kidney proximal tubule cells, thereby enhancing immune responses (38, 39). Among them, fatty acids β-oxidation (FAO) in renal proximal tubules is promoted by high-expression of PPAR-α in acute kidney injury (AKI) disease induced by cisplatin (CP) or ischemia/reperfusion (I/R). Thus, the lipoperoxides, including 4-hydroxy-2-hexenal, would be reduced by probiotics in koumiss, which help alleviate or hinder oxidative stress-mediated AKI. This also prevents cell apoptosis and proximal tubular cell death from occurring (40–42). Therefore, probiotics in koumiss can prevent kidney damage and protect kidney function (43, 44).

*Saccharomyces cerevisiae* isolated from koumiss can secrete antibacterial compounds, the main ingredients of which include citric acid, ascorbic acid, lactic acid, malic acid, and killer toxins (45, 46). These antibacterial compounds can effectively increase the concentration of immunoglobulin A (IgA), which can rapidly initiate the humoral immune response, and increase the number of beneficial bacteria while reducing the number of harmful bacteria or viruses in the gastrointestinal tract, together strengthening the resistance to various diseases (47–49). These antibacterial compounds in koumiss can also increase the concentrations of CD3+ and mature T lymphocytes in peripheral lymphoid organs to enhance the immune function of lymphoid organs or can decrease the concentrations of CD8+ in T lymphocytes to regulate the ratio of CD4+/CD8+ (50, 51). Humoral immunity is increased by antibacterial compounds and would be increased when infected by *Escherichia coli* O8 (51, 52). The antibacterial compounds can increase the numbers of *Bifidobacterium* and inhibit extraintestinal pathogenic *E. coli* in the cecum. Additionally, they can regulate the pH of the intestine and inhibit harmful bacteria by producing acetic acid. This helps maintain a normal microflora structure in the cecum, promoting physical health and preventing various diseases (51). Therefore, koumiss consumption can effectively strengthen the body’s immunocompetence and anti-inflammatory responses.

### Koumiss can effectively reduce the occurrence of cancer

The probiotics found in koumiss have been shown to strengthen the immune system, suppress tumor growth, and prevent the accumulation of carcinogenic compounds. Lactic acid bacteria is considered the dominant probiotic involved in the process of koumiss fermentation (53, 54). Additionally, it has been found that lactic acid bacteria isolated from koumiss collected in Xinjiang, China effectively reduced the number of colon cancer HT-29 cells. When *Limosilactobacillus reuteri* (*Lactobacillus reuteri*) BCRC14625 (1.0 × 10^9^ CFU/mL) was cultured together with HT-29 cells, there was a significant increase in lactate dehydrogenase (LDH) activity (*p* < 0.05), resulting in damage to the cell membrane. In contrast, *L. reuteri* BCRC14625 has been observed inducing HT-29 cancer cells to secrete nitric oxide (NO), leading to apoptosis of the cells. Therefore, koumiss may be both a potential preventive and therapeutic agent for treating colon cancer (55–57).

**Figure 2:**
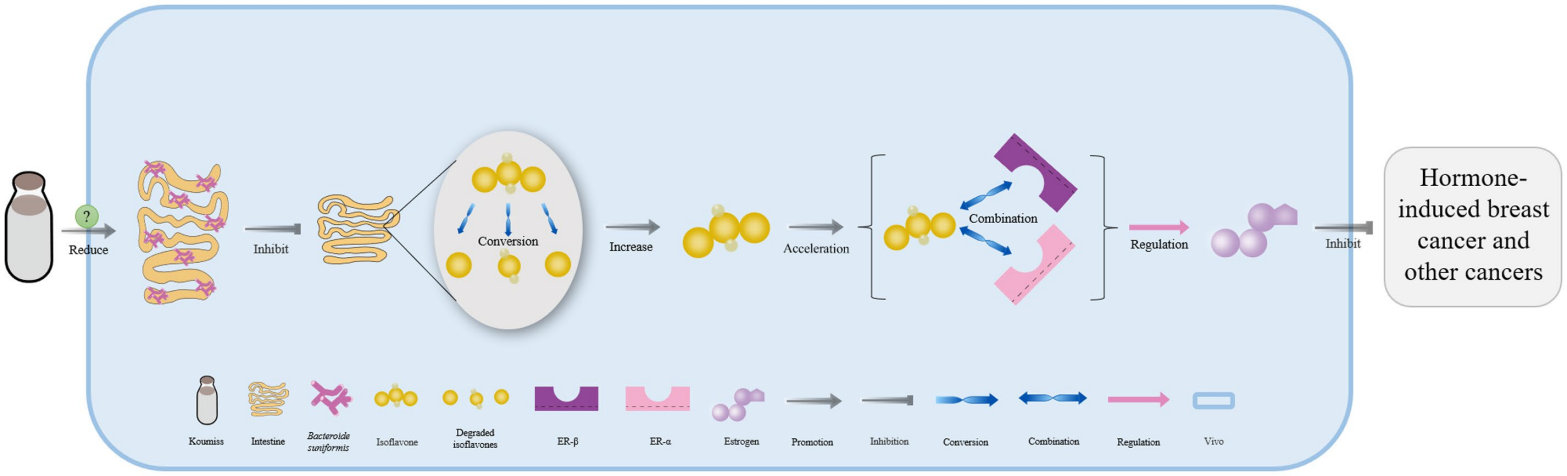
The mechanism of cancer prevention effectively with koumiss. Drinking koumiss can reduce the concentration of *Bacteroides uniformis in vivo* by some certain ways, which in turn inhibits the degradation of isoflavones in the intestine. This results in more isoflavones being available to accelerate binding to estrogen receptors (ER α and ER β) as estrogen-like compounds, directly regulating the level of estrogen *in vivo* to prevent hormone-induced breast cancer and other cancers.

Long-term consumption of koumiss had been shown to reduce *Bacteroides uniformis* concentrations, which are responsible for degrading isoflavones *in vivo* (*p* = 0.016). This results in a greater concentration of isoflavones that can bind to estrogen receptors (ERα and ERβ) as estrogen-like compounds in patients. Therefore, the level of estrogen in the body can be regulated to prevent hormone-induced breast cancer and other cancers (Figure 2) (58–60). Drinking koumiss can potentially serve as an early predictor of gastric cancer, while also relieving the symptoms of chronic atrophic gastritis. Furthermore, consuming koumiss can significantly reduce blood platelet counts, which in turn, may prevent inflammation and cancer (60–63).

Interferon γ (IFN- γ) levels can be increased greatly *in vivo* by *L. casei* Zhang isolated from koumiss (28). IFN-γ plays an important role in the antiproliferation of ovarian cancer, rectal cancer, and hepatocellular carcinoma. High doses of IFN-γ have been shown to promote the transcription and synthesis of protease caspase 3 and caspase 7. These activate the apoptosis process of cancer cells by initiating Janus kinase-signal transducers and activators of the transcription 1-caspase (JAK-STAT1-caspase) signal (32, 64, 65). Research has shown that IFN-γ can downregulate the expression of vascular endothelial growth factor A (VEGFA), which in turn disturbs the proliferation and survival of endothelial cells (66). This leads to blocking angiogenesis in the tumor microenvironment, ultimately preventing tumor growth. IFN-γ can also block the interleukin 8-chemokine receptor CXCR2 (CXCL8-CXCR2) axis, which prevents the timely transportation of CXCR2+ CD68+ immunosuppressive macrophages to the tumor microenvironment (TME). This enhances the therapeutic effects of programmed cell death protein 1 (PD-1) blockade therapy for pancreatic cancer (67).

Consuming koumiss can also significantly reduce the levels of lithocholic acid and bile acid in feces, which can help prevent intrahepatic cholestasis and reduce liver cancer incidence (68–71). These findings demonstrate that the probiotics found in koumiss should be effective in reducing the incidence rates of tumors and cancers.

### Koumiss can effectively reduce *in vivo* cholesterol levels

The results of high throughput sequencing have shown that lactic acid bacteria is the dominant flora present in koumiss. Through assimilation of lactic acid bacteria, cholesterol in the cell membrane can be bound to the phospholipid tail, upper-phospholipid, and polar head regions, and thereby be converted to coprostanol and reduce cholesterol levels *in vivo* (60, 72–74). The binding of lactic acid bacteria with bile acids can inhibit its absorption by the small intestine and facilitate its excretion in feces. Endogenous cholesterol is then sequentially converted into bile acid, ultimately leading to a reduction in cholesterol levels (74, 75). Additionally, the contents of short-chain fatty acids (SCFAs) such as propionate and butyrate in the intestine increases in the presence of three lactic acid bacteria strains (*Lactobacillus helveticus* MG9-2, *Lactiplantibacillus plantarum* LIP-1, and *Limosilactobacillus fermentum* E7301) isolated from koumiss. The activity of pyruvate dehydrogenase in the liver is effectively restrained thereby leading to the inhibition of fatty acid synthesis and ultimately resulting in a reduction in the concentration of cholesterol in serum and the liver (74, 76). lactic acid bacteria in koumiss can also effectively increase high-density lipoprotein in the blood, which can reduce or block the flow of cholesterol into the liver (77, 78).

**Figure 3:**
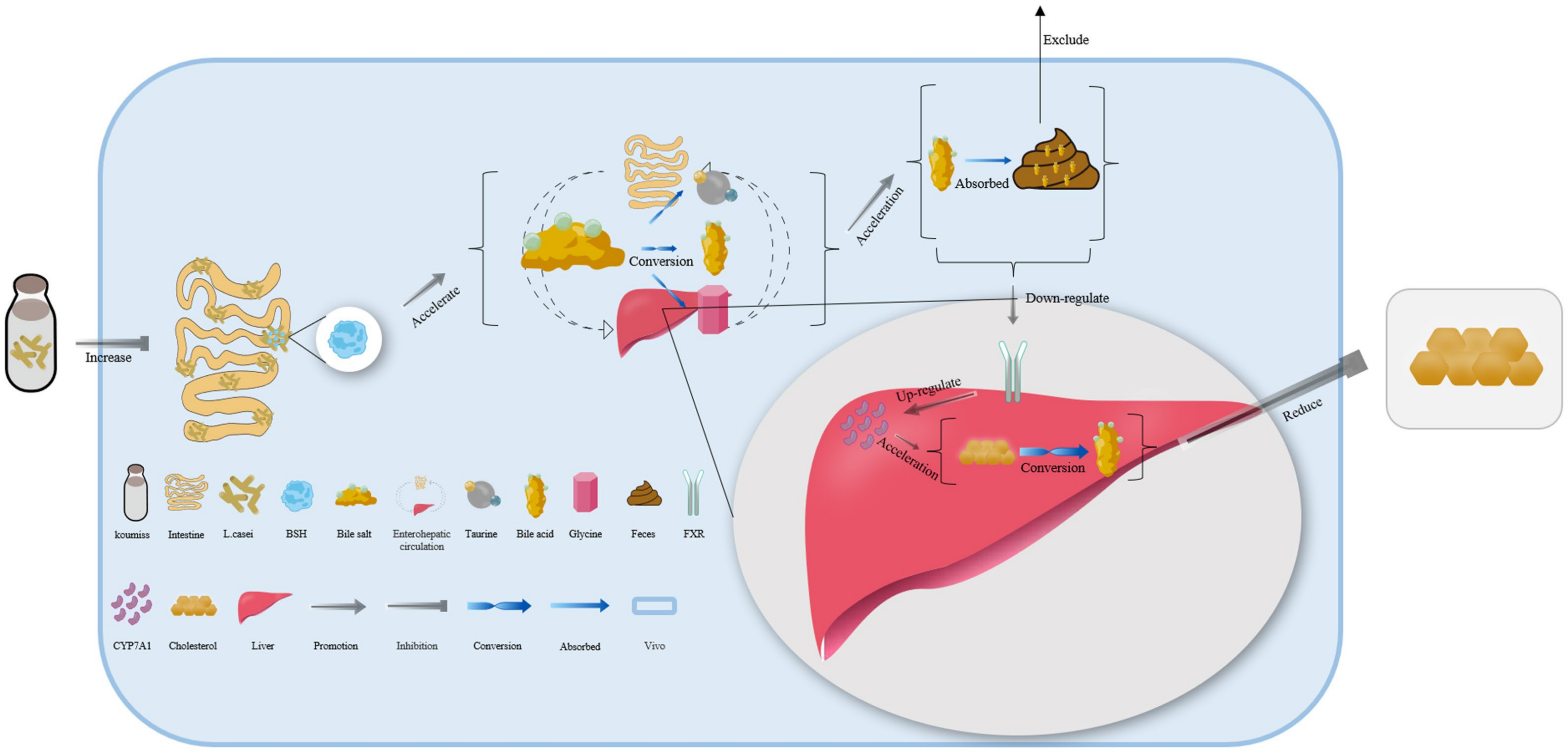
The mechanism of cholesterol content reduction with koumiss *in vivo*. Drinking koumiss can increase the content of bile salt hydrolase (BSH) secreted by *Lacticaseibacillus casei*, which can accelerate the conversion of bile salts into bile acid, taurine, or glycine in enterohepatic circulation. The excessive bile acid can be absorbed by the feces and excreted out of the body. Furthermore, the requirements of bile acid would down-regulate the expression level of farnesoid x receptor (FXR) and up-regulate CYP7A1, accelerating the conversion of cholesterol to bile acid. This, in turn, significantly lowers the content of cholesterol levels *in vivo*.

*Lacticaseibacillus casei* in koumiss collected from Xinjiang has been shown to secrete bile salt hydrolase, which catalyzes the deconjugation of bile salts in enterohepatic circulation (55). This leads to the release of glycine or taurine groups from the steroid’s core molecular structure, converting conjugated bile acid into free bile acid that is excreted in feces. As a result, the bile acid content in the body is reduced (Figure 3) (79, 80). Additionally, the expression of the farnesoid x receptor is significantly downregulated, while that of cholesterol 7-alpha hydroxylase (CYP7A1) is upregulated, both of which can accelerate the conversion of bile acid from endogenous cholesterol in the liver. This ultimately helps maintain normal metabolic activities (Figure 3) (81–83).

A total of seven metabolites naturally found in koumiss have been identified in the feces of patients with hyperlipidemia who had been drinking koumiss for an extended period. These metabolites include stearic acid, sphingosine, tyrosine, α-tocotrienol, γ-tocotrienol, butyric acid, and butyrate (84). Stearic acid, a saturated fatty acid, can increase the oxidation rate of low-density lipoprotein (LDL) cholesterol in the blood after being converted to oleic acid (85). Sphingosine is bound to cholesterol to form a complex that can limit intestinal cholesterol absorption. Sphingosine can also attenuate the affinity of transporter Niemann Pick C1 like 1 (NPC1L1) toward cholesterol (86). Additionally, tyrosine can be converted to adrenaline, which further promotes the process of lipid hydrolysis *in vivo* (87), and α-tocotrienol and γ-tocotrienol downregulate the expression of HMG CoA reductase to inhibit the synthesis of endogenous cholesterol and linoleic acid promotes β-oxidation, thus reducing the synthesis of endogenous triglycerides (TGs). Butyrate accelerates the hydrolysis of fatty acids, leading to a reduction in the concentration of TGs in the blood, and can also block the transportation of Very Low-Density Lipoprotein Cholesterol (VLDL-C) from the liver to the blood (84). Therefore, consuming koumiss can help reduce cholesterol through multiple processes.

### Koumiss treats chronic gastritis and regulates intestinal flora

**Figure 4:**
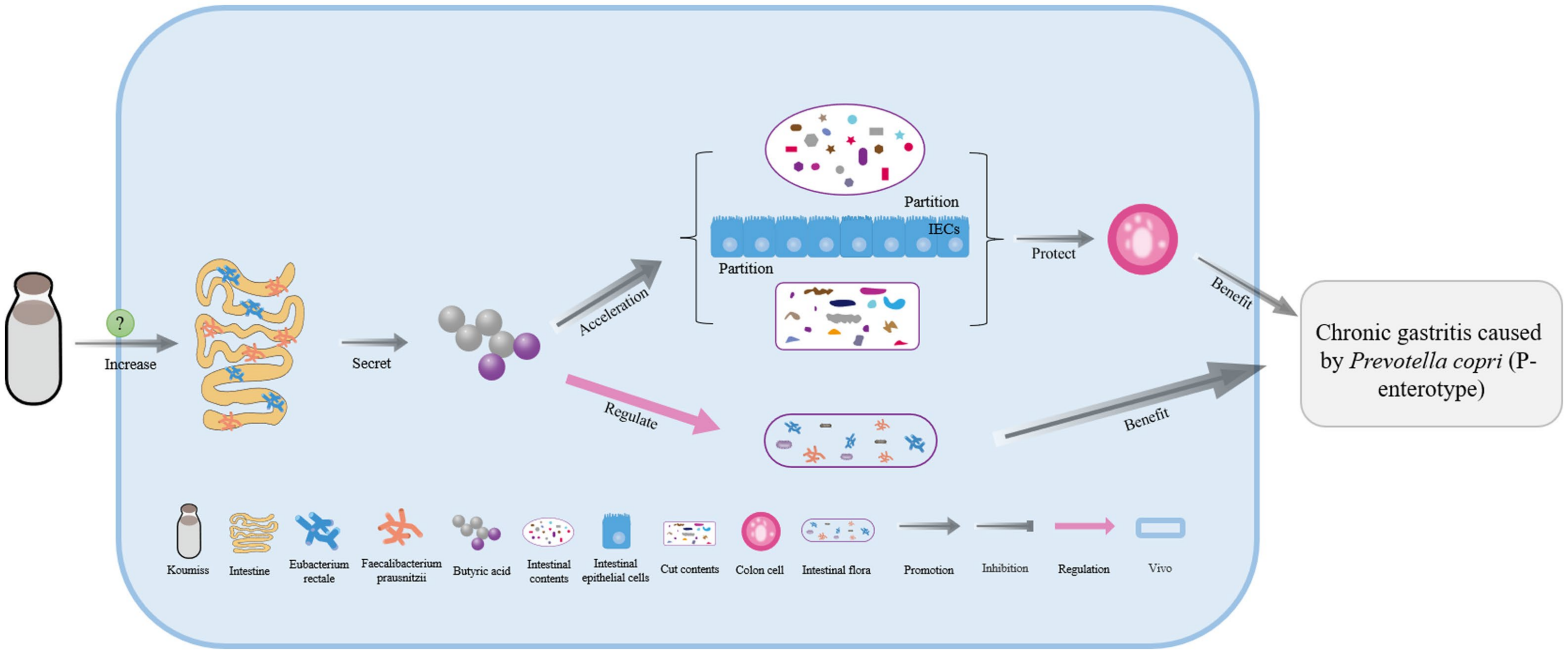
The mechanism of chronic gastrotis improvement with koumiss. Drinking koumiss can significantly increase the numbers of *Eubacterium rectale* and *Faecalibacterium prausnitzii* in the intestine by some certain ways, which can secrete more butyric acid to regulate the intestinal flora and help the intestinal and gut contents separated by intestinal epithelial cells (IECs) and then protect the colonic epithelial cells. As a result, chronic gastritis caused by *Prevotella copri* (P-enterotype) can be effectively treated.

Koumiss is a functional drink that can be used to treat chronic gastritis. Studies have shown that when mice consumed koumiss, their intestinal numbers of *Eubacterium rectale* and *Faecalibacterium prausnitzii* significantly increased (60). These two intestinal microorganisms secrete butyric acid, which can regulate intestinal flora disorders and effectively promote gut content isolation and hypodermis among intestinal epithelial cells, thus stabilizing colon cells. These actions can inhibit the growth of cancer cells and reduce inflammation, making koumiss an effective treatment for chronic gastritis caused by *Prevotella copri* (enterotype 2) in the gut (Figure 4) (60, 88).

Koumiss had also been shown to be effective in treating gastrointestinal inflammation caused by *Salmonella typhimurium* ATCC 14028 (89). Consuming koumiss can boost the relative abundance of *Akkermania muciniphila* and Lachnospiraceae in the intestine. Autonomic control of systemic glucose metabolism and energy balance of *A. muciniphila* can inhibit the growth and reproduction of *Toxoplasma gondii* in the intestinal tract. SCFAs produced by *Lachnospiraceae* not only stimulated the proliferation of colon epithelial cells and reduce colonic transit time but also provide nutrients for the colon mucosa, preventing mucosal atrophy. By maintaining primal gastrointestinal morphology and normal physiological functioning, koumiss provides an effective treatment option (90–92).

### Other health functions of koumiss

**Figure 5:**
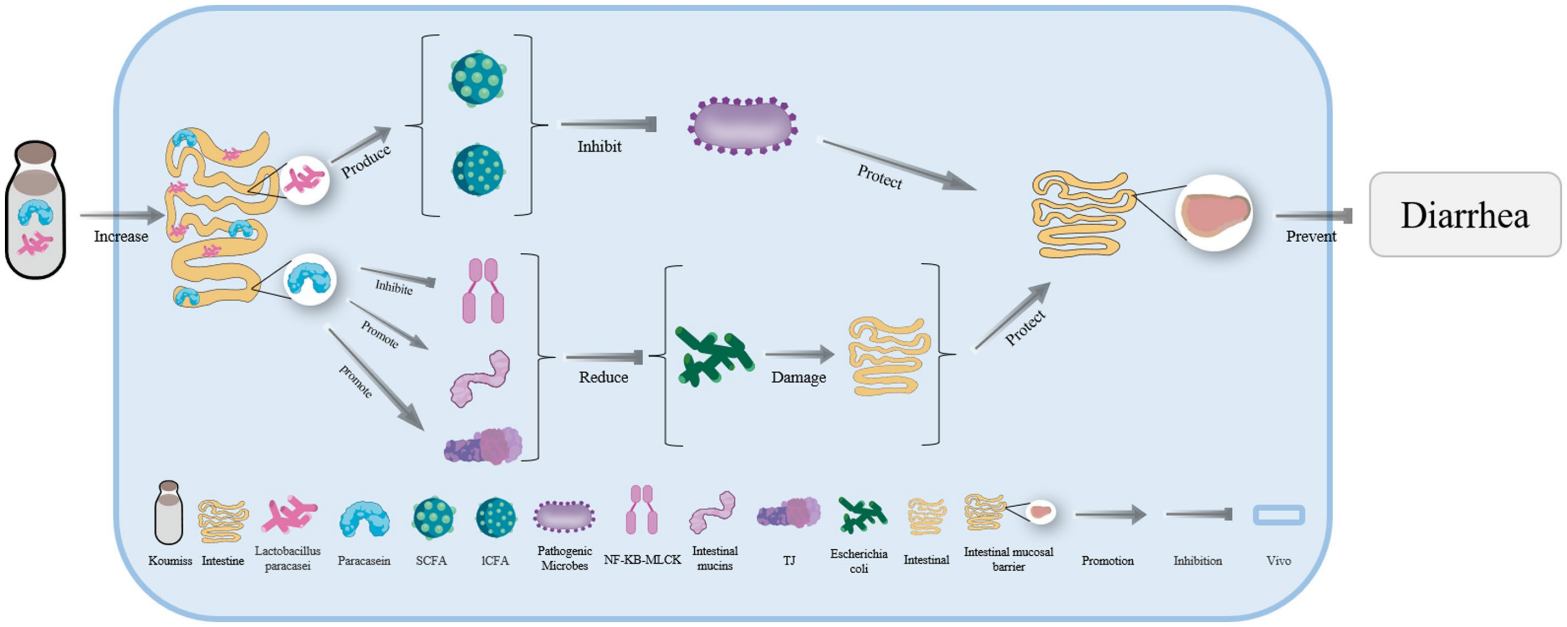
The mechanism of diarrhea treatment with koumiss. Drinking koumiss can enhance the number of *Lacticaseibacillus paracasei* and paracasein *in vivo*. SCFA and lCFA are produced by *L. paracasei* to inhibit the reproduction of pathogenic microorganisms. On the other hand, paracasein can catalyze the secretion of intestinal mucins and TJ while inhibiting the transmission of NF-KB-MLCK, reducing the damage of the intestine by *Escherichia coli*. This, in turn, helps maintain the integrity of the intestinal mucosal barrier (IMB) and prevents diarrhea.

A large number of *Lacticaseibacillus paracasei* and abundant paracaseins are also found in koumiss (Figure 5). Some substances, such as short-chain or long-chain fatty acids produced by *L. paracasei* can effectively inhibit the reproduction of pathogenic microorganisms (Figure 5). Paracaseins can induce endogenous mucin secretion, promote the expression of tight junction proteins, and inhibit the transmission of the NF-kB MLCK signal pathways (Figure 5). As a result, the intestinal damage caused by *E. coli* can be reduced and the intestinal mucosal barrier can be strengthened to effectively treat diarrhea (Figure 5) (93).

Consumption of koumiss can increase the numbers of *Vibrio desulfovibrio* in mouse guts and generate more H_2_S. This can lead to the disordered oxidation pathway of butyric acid in cells, resulting in DNA chain breakage, inhibition of ATP-dependent K^+^ channels, and the release of cytochrome C which can help reduce both pulmonary and extrapulmonary tuberculosis in hosts (94, 95). It has also been reported that drinking koumiss can prevent tuberculosis caused by the pathogen *Mycobacterium tuberculosis* H37RV (89).

Consuming koumiss has been shown to effectively repair glomerular damage and prevent chronic liver and kidney diseases by increasing platelet-derived growth factor c (PDGF-c) and platelet-derived growth factor receptor alpha (PDGFR-α) expression (38, 96). Additionally, *L. casei* Zhang in koumiss downregulates the expression of toll-like receptors 4 (TLR4) in the liver, promotes the production of TNF-α and oxidative stress responses, and effectively protects the liver (97). Furthermore, koumiss contains angiotensin-converting enzyme inhibitors, which can reduce Angiotensin II (Ang II) generation and reduce hypertension (98–100).

## Microbial community alternation during koumiss fermentation

It has been found that total of 12 phyla, 124 genera, and 227 species were found across 29 koumiss samples while 18 phyla, 286 genera, and 491 species across 13 fresh mare milk samples (101). Alghough fresh mare milk is rich in LAB, such as *Lactobacillus helveticus*, *Lactiplantibacillus plantarum*, *L. kefiranofaciens* and *Lactococcus lactis*, potential microbiological hazards can not be ignored as well. Variable levels of total microbial count (TMC) are 3.40–5.87 (log CFU/ml) in the fresh mare milk, including some potentially pathogens such as *Acinetobacter*, *Klebsiella*, *Escherichia*, *Brucellosis*, *Campylobacter*, *Mycobacteriosis*, *Bacillus*, *Staphylococcus*, *Streptococcus Cronobacter*, and others (10). However, the koumiss microbiota mainly comprised LAB instand of potential pathogens, and sequences representing pathogenic bacteria were not detected (101). In koumiss, *Lactobacillus* (such as *Lentilactobacillus diolivorans*, *Lactobacillus acidophilus*, *Lacticaseibacillus casei*, *Latilactobacillus curvatus*), *Streptococcus*, and *Saccharomyces cerevisiae* are still the dominated the microbial community as the starter (102, 103). Therefore, the bacterial microbiota diversity of the koumiss was more simplex than the fresh mare milk. In spite of these researches, lower abundance of potential pathogens would be also living in the koumiss during the natural fermentation process and would become the microbiological hazards that should be further assessed with accurate methods.

## Conclusions and future perspectives

Koumiss, a traditional fermented dairy product with a long production history, is rich in nutrients such as vitamins C and E, has a balanced calcium-phosphorus ratio, and contains various beneficial trace elements. The natural fermentation process of koumiss leads to the formation of a unique flora structure consisting of a large variety of probiotic compounds. These produce organic acids, extracellular polysaccharides, functional peptides, and other metabolites. Therefore, koumiss can be regarded as a medicinal food that provides a series of special health functions, including immunity enhancement, cancer and tumor prevention, cholesterol reduction, chronic gastritis treatment, and intestinal flora regulation, among others. But, we should be pay attention to the potential pathogens in koumiss. So, standardized koumiss fermentation process should be established with the excellent probiotics as the starter to control the growth and reproduction of harmful bacteria in the future.

Although the special health functions of koumiss have been confirmed through limited animal testing, further studies are needed to elucidate the molecular mechanisms that enable its preventive and therapeutic effects on human diseases. This involves isolating and identifying many unique probiotics present in koumiss for more specialized applications. Furthermore, the molecular regulatory mechanisms underlying the alteration of intestinal flora, as well as other treatments using the probiotics and their metabolites found in koumiss, must be further explored by researchers. In contrast, harmful metabolites produced by these potential pathogens in koumiss should be distinguished by metabolomics technology. And the clinical allergies, intolerances, or contraindications of koumiss should be studied furtherly.

## Author contributions

WX: Writing – original draft. XY: Funding acquisition, Conceptualization, Writing – review & editing. ZJ: Funding acquisition, Writing – review & editing. HL: Funding acquisition, Writing – review & editing. YY: Conceptualization, Writing – original draft.
